# Supplementary material for: The structure of the bacterial DNA segregation ATPase filament reveals the conformational plasticity of ParA upon DNA binding
Source: Nat Commun. 2021 Aug 27;12:5166. doi: 10.1038/s41467-021-25429-2 (PMC8397727; doi:10.1038/s41467-021-25429-2)
Supplement: Supplementary file 1 — Supplementary Information [file 41467_2021_25429_MOESM1_ESM.pdf]

SUPPLEMENTARY FIGURES

The structure of the bacterial DNA segregation ATPase filament reveals the conformational plasticity of ParA upon DNA binding.

Parker AV[1], Mann D[1,4], Tzokov SB[1], Hwang LC[1,2]*, Bergeron JRC[1,3]*

1 Department of Molecular Biology and Biotechnology, the University of Sheffield, UK

2 Current address: School of medicine, Faculty of Health, Education, Medicine and Social Care, Anglia Ruskin University, UK

3 Randall Centre of Cell and Molecular Biophysics, King's College London, UK

4 Current address: Ernst-Ruska centre 3, Forschungszentrum Jülich, Germany

* Correspondence to: ling.hwang@aru.ac.uk or julien.bergeron@kcl.ac.uk

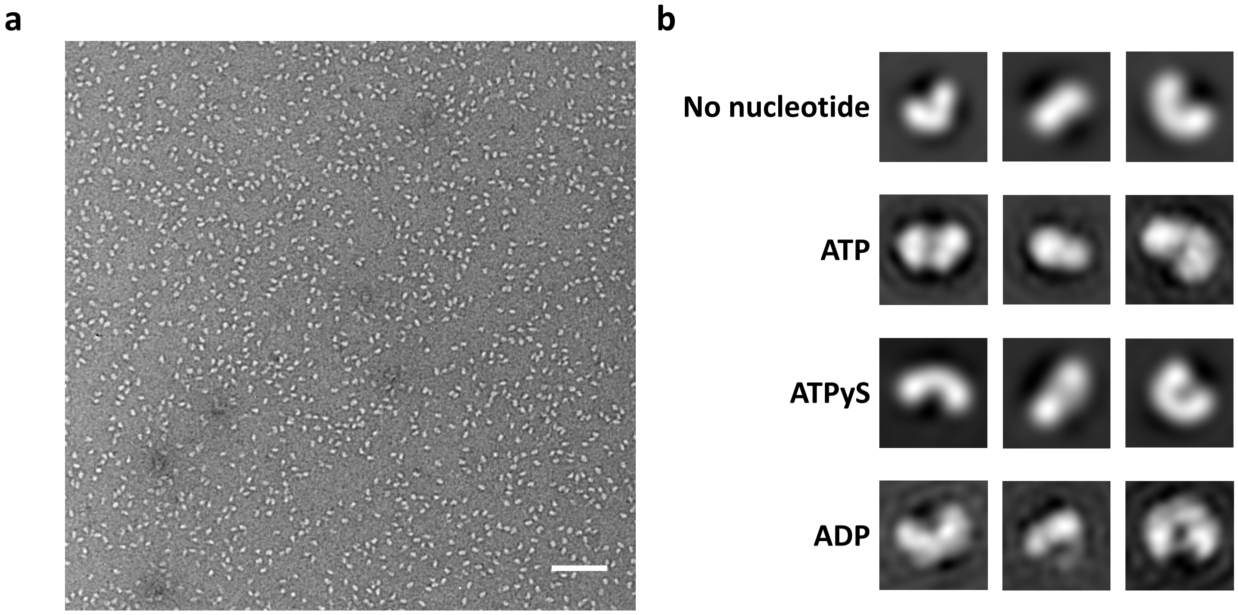

Supplementary Figure 1: Micrograph and 2D classes of ParA2$_{vc}$ dimers. (a) Representative negative-stain electron micrograph (out of a 10-20 micrograph dataset) of ParA2$_{vc}$ in the presence of ATP. Elongated particles, approximately 10 nm long, are visible. The scale bar indicates 100nm. **(b)** 2D classes of particles from negative-stain EM micrographs of ParA2$_{vc}$ in varying nucleotide states. Two-lobbed particles, consistent with a dimer in different orientations, are visible in all states.

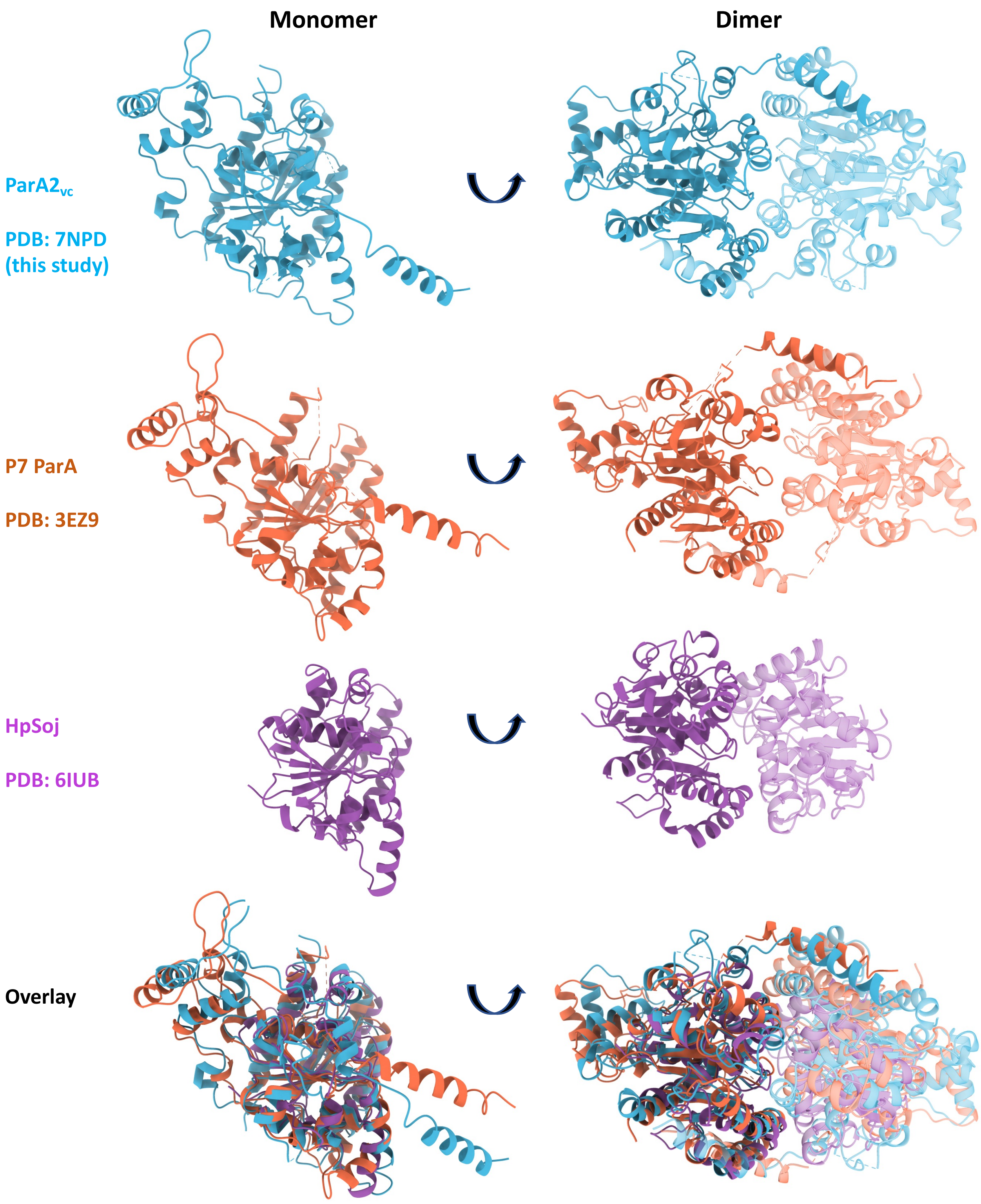

Supplementary Figure 2: Comparison of ParA structures across bacterial species. A monomer (left) and dimer (right) is shown for the structures of ParA2$_{VC}$ (cyan), the *E.coli* P7 plasmid ParA (orange), and $_{Hp}$Soj (purple). All three structures are aligned at the bottom, on the left chain for the dimer.

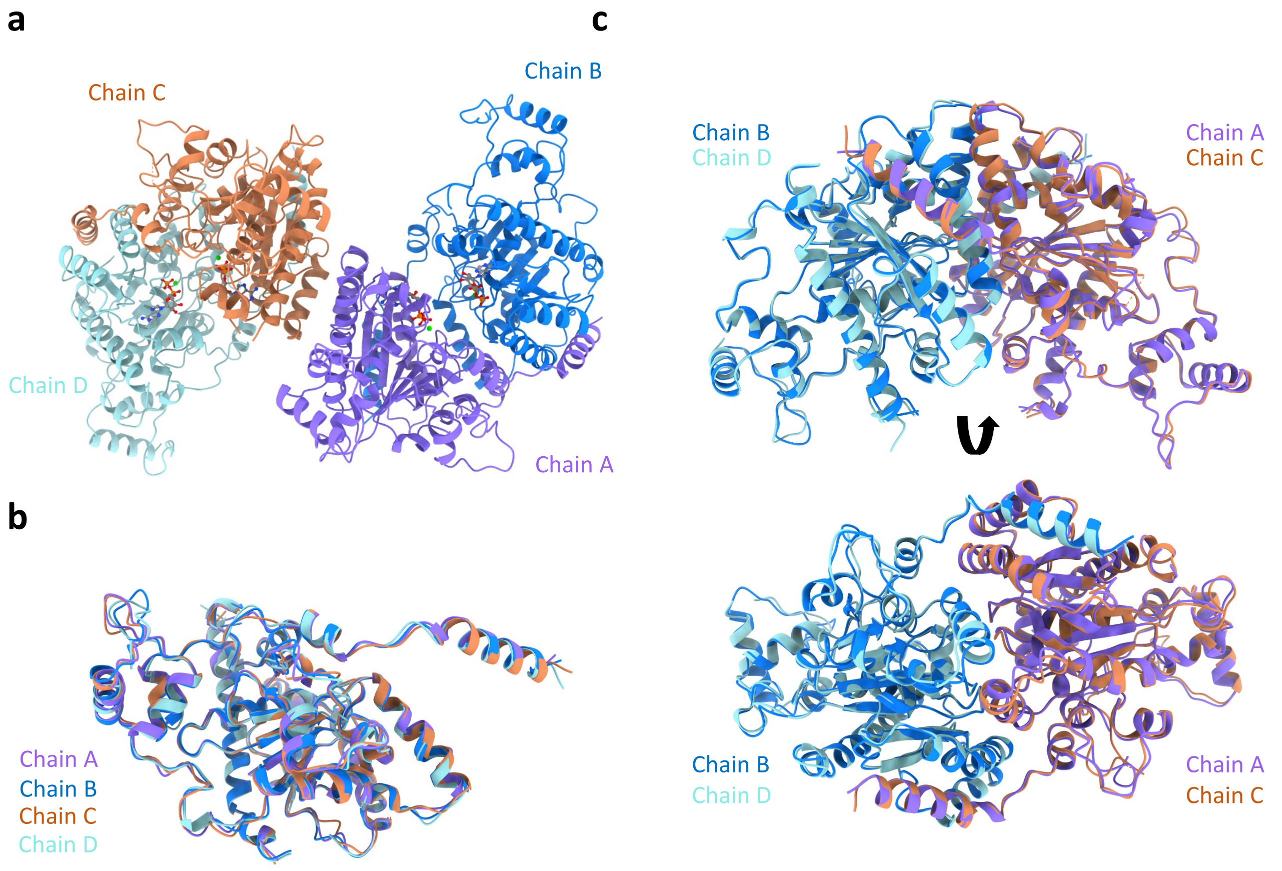

Supplementary Figure 3: Comparison of chains in the ParA2$_{vc}$-ADP asymmetric unit. **(a)** Cartoon representation of the ParA2vc-ADP structure asymmetric unit, showing 4 protein molecules, in purple, blue, orange, and cyan, respectively. Density for the nucleotide is found in all four. **(b)** Overlay of all 4 chains, coloured as in (a). **(c)** Side (top) and top (bottom) views of the two dimers in the ASU, coloured as in (a). All four chains only show minor variations in loop regions, and the dimeric architecture is the same for the two dimers.

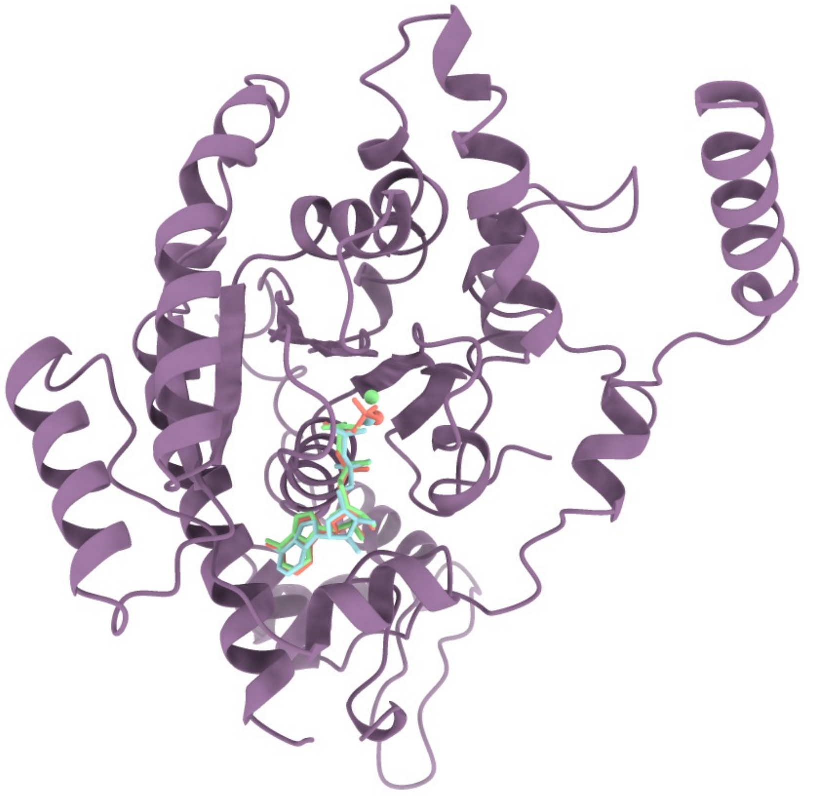

Supplementary Figure 4: Nucleotide binding in ParA orthologues. The structure of $ParA2_{vc}$ is shown in purple, with the bound ADP molecule in cyan. The nucleotide in overlaid P1 ParA structure (ADP:PDB ID 3ZE6) and in hpSoj (ATP:PDB ID 6IUB) are in green and orange, respectively. The nucleotide is positioned similarly in all three structures.

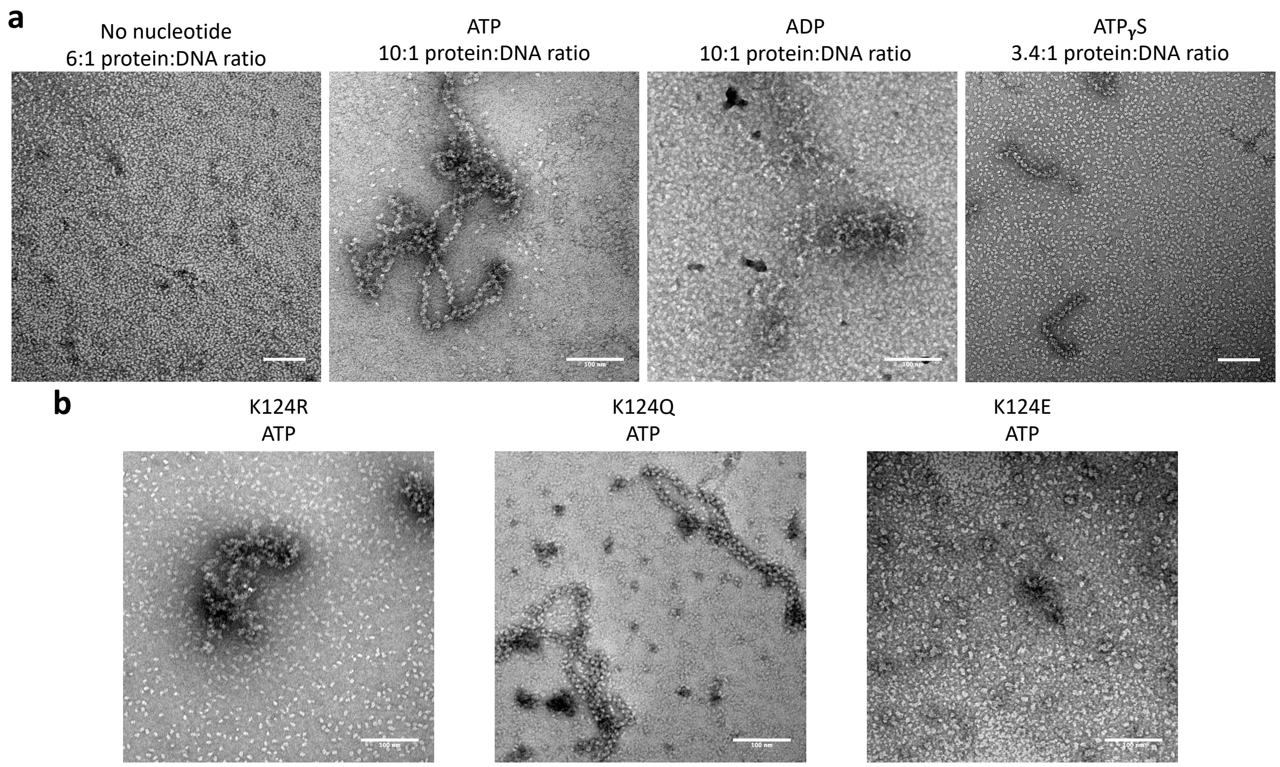

Supplementary Figure 5: Negative-stain electron micrographs of ParA2$_{vc}$ with ssDNA, in varying nucleotide states. All micrographs are representatives of 10-20 micrograph datasets. (**a)** Filament formation in various nucleotide state. The white bar indicates 100 nm. ParA2$_{vc}$ only forms filaments on DNA in the presence of nucleotide, and in large excess of protein. At lower protein concentration, ATP$_\gamma$S is required to observe stable filaments. **(b)** Filament formation with mutations that affect nucleotide binding/hydrolysis. The conservative mutations K124R and K124Q still permit filament assembly, but no filaments were observed with the more deleterious K124E mutation.

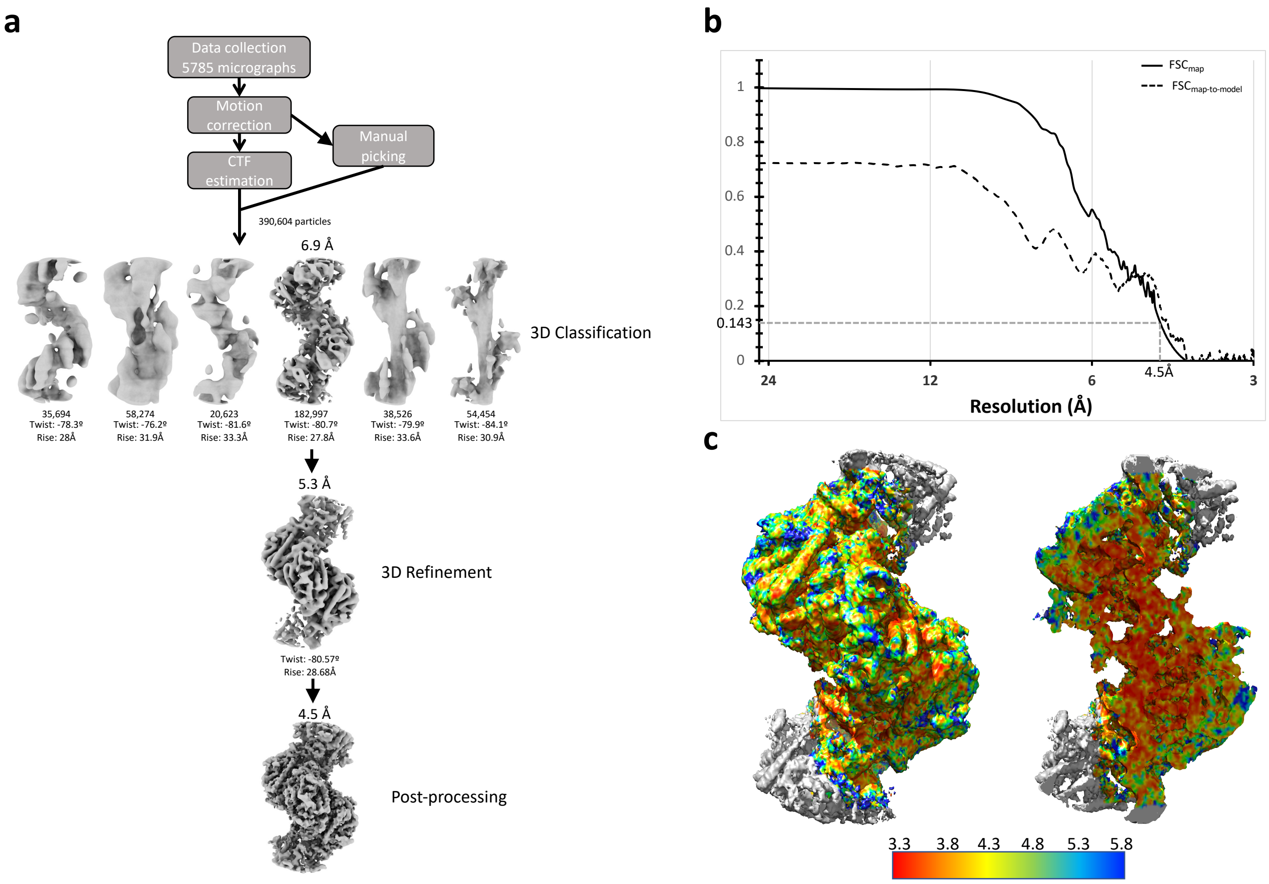

Supplementary Figure 6: Details of the ParA2$_{vc}$-ATPγS-DNA filament cryo-EM structure determination. (a) Data processing pipeline. **(b)** FSC curves for the masked post-processing refinement. **(c)** Local resolution of the electron potential map, calculated using ResMal with the scale bar in Å.

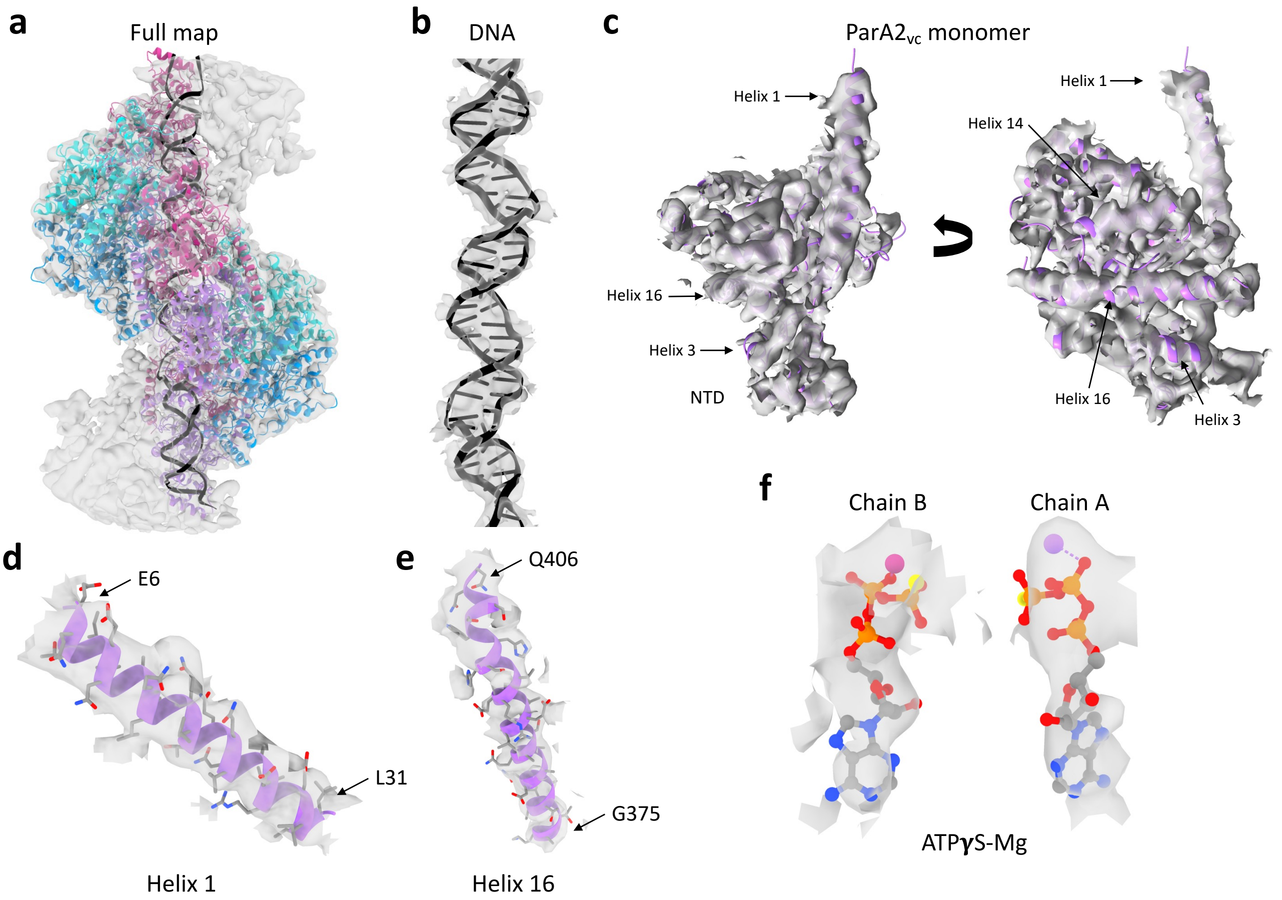

Supplementary Figure 7: Examples of the fit of the ParA2$_{vc}$-ATPγS-DNA atomic model to the electron potential map. Various elements are shown, including the full atomic model **(a)**, DNA **(b)**, a single ParA2$_{vc}$ monomer **(c)**, the N-terminal **(d)** and C-terminal **(e)** helices, and the two nucleotides of the central dimer **(f)**. These illustrate that the map features are consistent with the reported resolution.

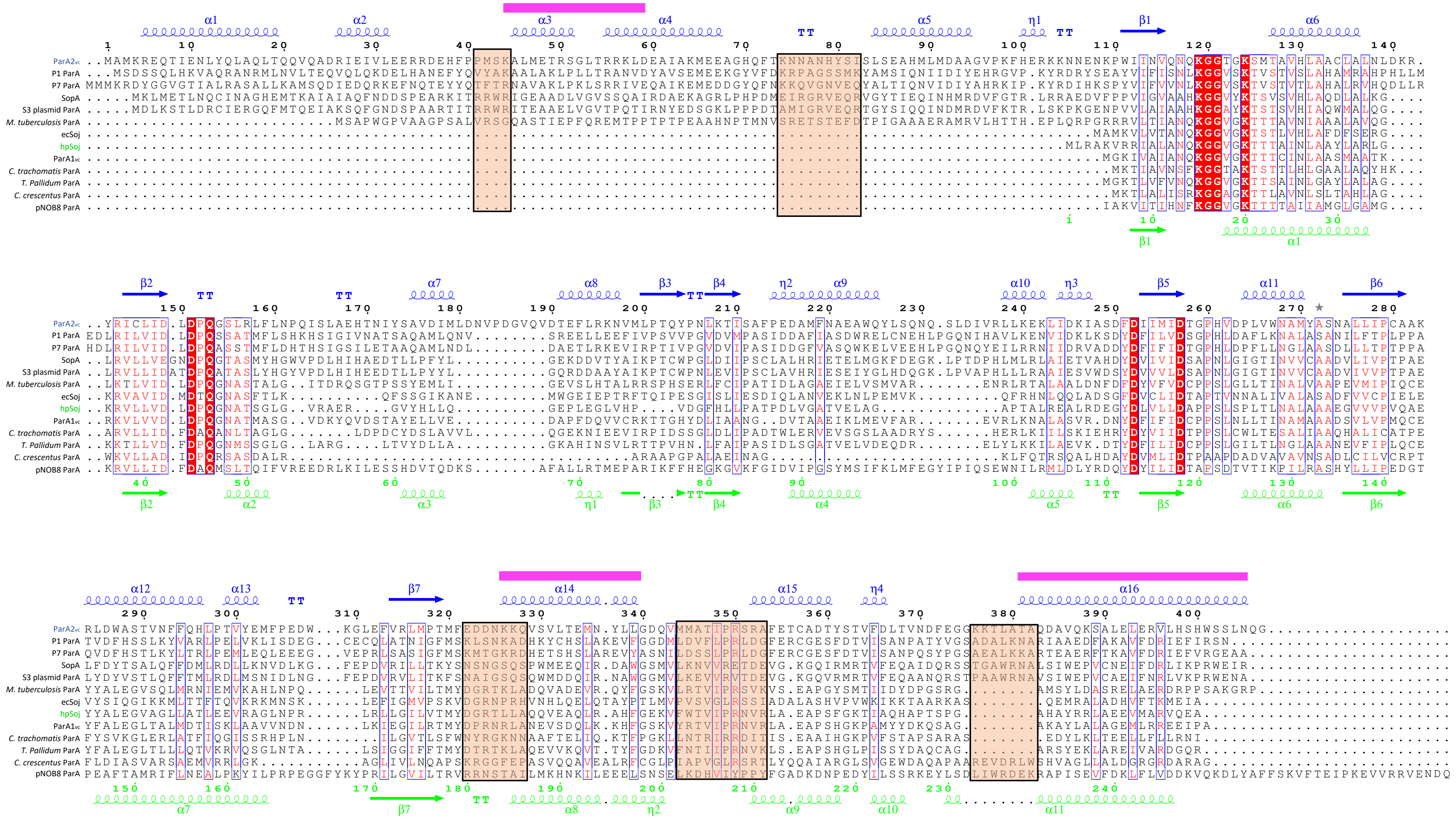

Supplementary Figure 8: Multiple sequence alignment of various ParA orthologues. The first six are Type Ia sequences, the next seven are type Ib sequences, and the bottom sequence is for the archaea pNOB8 plasmid ParA. The secondary structure elements for $ParA2_{vc}$ is in blue at the top, and for HpSoj in green at the bottom. Conserved residues are in red box, similar residues in red characters. The orange boxes indicate regions of $ParA2_{vc}$ that interact with DNA, and the purple bars indicate the filament oligomerization interfaces.

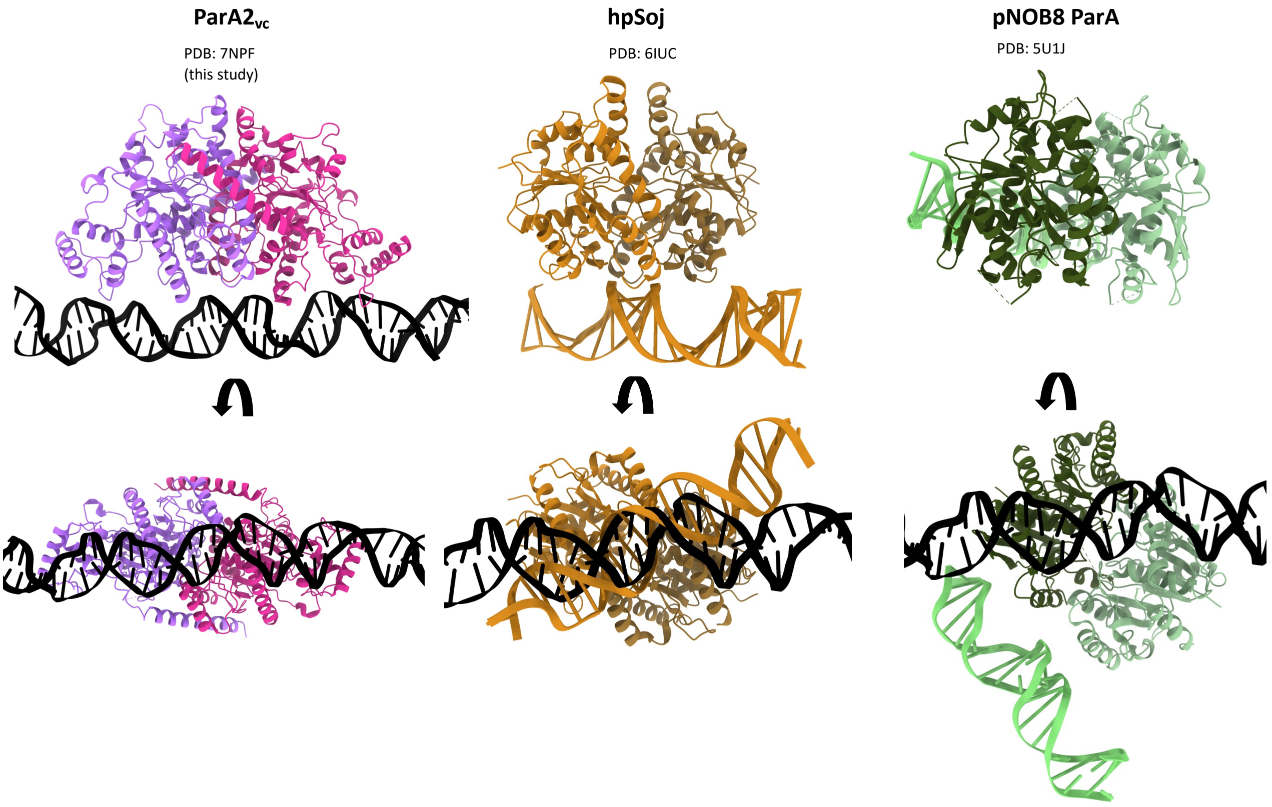

Supplementary Figure 9: Comparison of the DNA binding in ParA orthologues. The structure of the ParA dimer, from the side and bottom, is shown for ParA2$_{vc}$ (left), HpSoj (centre), and pNOB8 ParA (right). The two ParA2$_{vc}$ molecules are colored as in figure 3c, with the DNA in black. For HpSoj and pNOB8 ParA, the position of the DNA in the filament structure reported here is shown in black, for comparison.

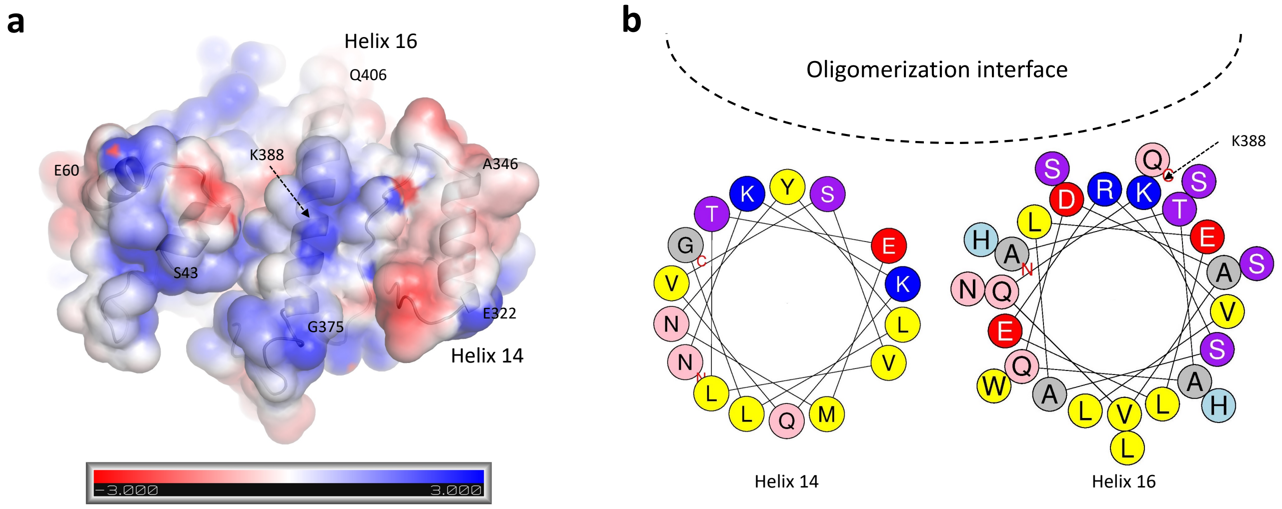

Supplementary Figure 10: Molecular details of the oligomerization interface. (a) surface representation of a ParA2$_{vc}$ monomer in the filament structure, colored by charge. The boundaries of the domains involved in dimer-dimer interaction are indicated. **(b)** Helical wheel plot of **α**-helices 14 and 16 of ParA2$_{vc}$, that form the filament oligomerization interface. The charged residues cluster on the interface side.

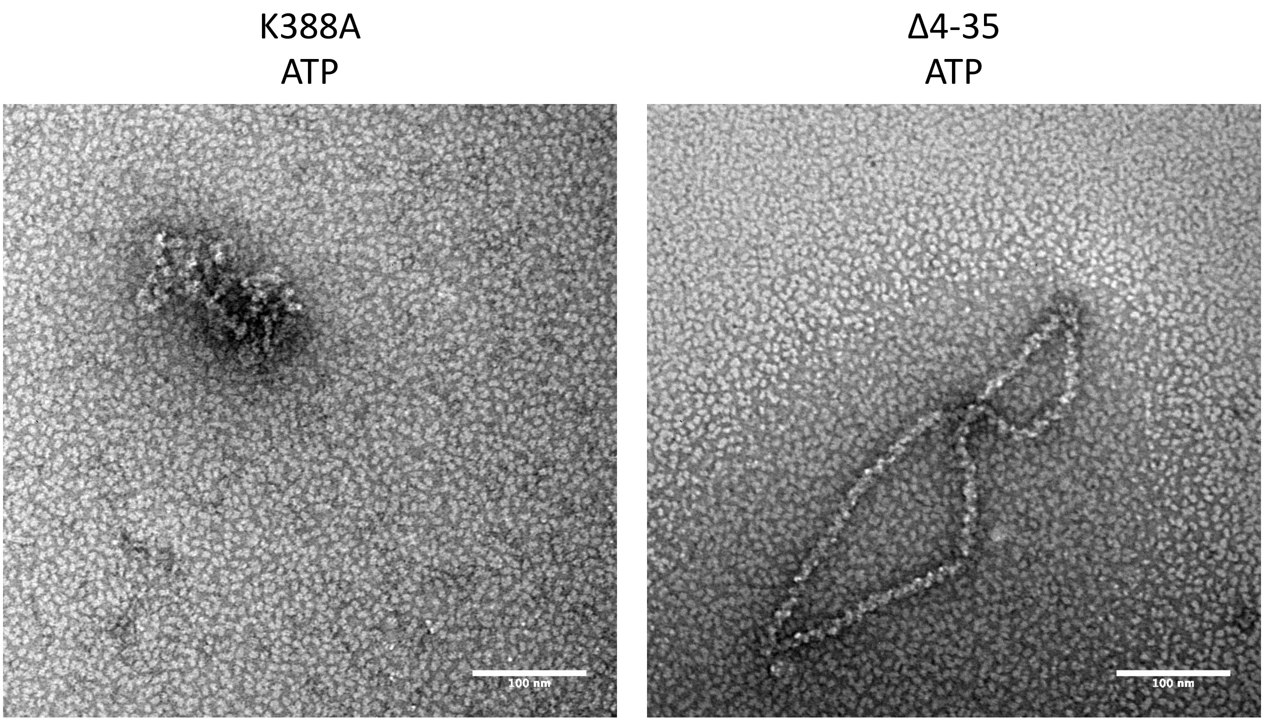

Supplementary Figure 11: Effect of the filament interface mutants of filament formation. No filaments are observed in the context of a point mutation at the filament interface (K388A). In contrast, deletion of the N-terminal helix of ParA (Δ3-36, left) does not impair its ability to form filaments. The scale bar indicates 100 nm.

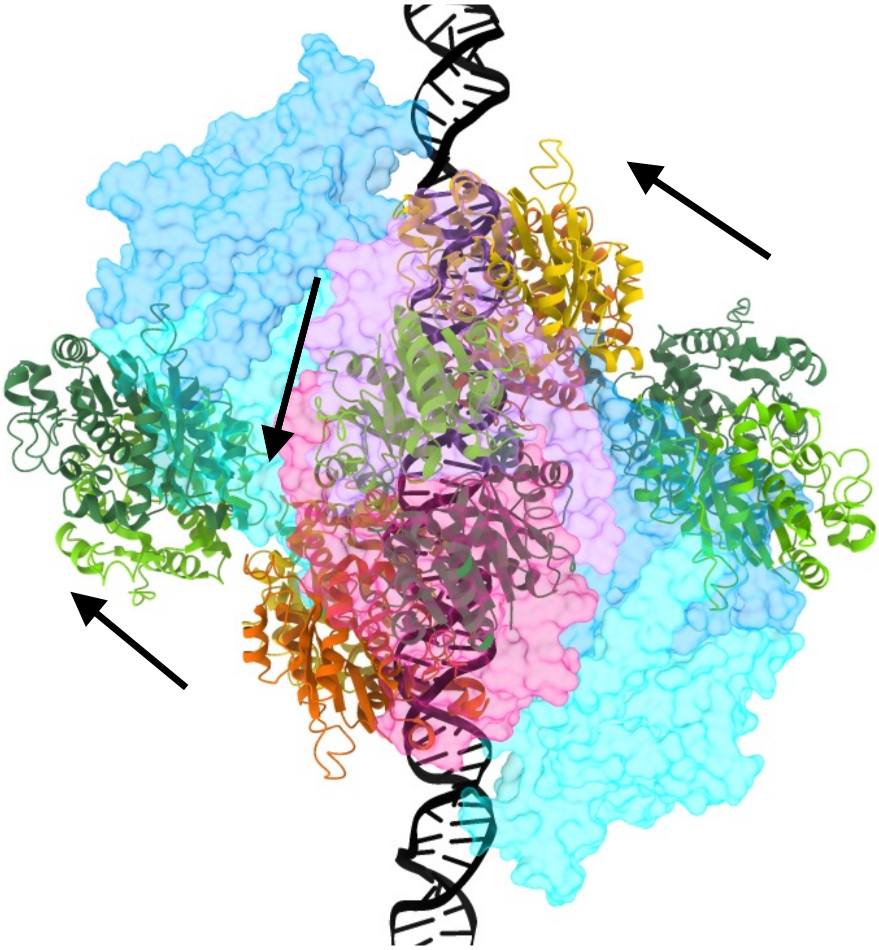

Supplementary Figure 12. Comparison of the ParA2$_{vc}$-DNA filament to MinD in the MinCD filament. A ParA2$_{vc}$ dimer bound to the DNA from the filament structure is shown, represented as in figure 3c. Adjacent MinD dimers from the MinCD filament structure are in light and dark green for one dimer, yellow and orange for the other. The central green MinD dimer is aligned to ParA2$_{vc}$, the arrows indicate the direction of the MinD filament architecture.
