# Supplementary material for: The structure of the bacterial DNA segregation ATPase filament reveals the conformational plasticity of ParA upon DNA binding
Source: Nat Commun. 2021 Aug 27;12:5166. doi: 10.1038/s41467-021-25429-2 (PMC8397727; doi:10.1038/s41467-021-25429-2)
Supplement: Supplementary file 7 — Description of additional supplementary files [file 41467_2021_25429_MOESM7_ESM.docx]

Description of additional supplementary files

Title: Supplementary movie 1

Description: Fit of the atomic model ito the ParA2vc-ATP𝛄SDNA cryo-EM map.

Title: Supplementary movie 2

Description: ParA2vc-DNA interaction, and filament interface.

Title: Supplementary Movie 3

Description: Structural changes of ParA2vc upon filament formation.
