# Supplementary material for: The structure of the bacterial DNA segregation ATPase filament reveals the conformational plasticity of ParA upon DNA binding
Source: Nat Commun. 2021 Aug 27;12:5166. doi: 10.1038/s41467-021-25429-2 (PMC8397727; doi:10.1038/s41467-021-25429-2)
Supplement: Supplementary file 8 — Reporting Summary [file 41467_2021_25429_MOESM8_ESM.pdf]

nature research

Corresponding author(s): Julien Bergeron

Last updated by author(s): Aug 2, 2021

Reporting Summary

Nature Research wishes to improve the reproducibility of the work that we publish. This form provides structure for consistency and transparency in reporting. For further information on Nature Research policies, see our Editorial Policies and the Editorial Policy Checklist.

Statistics

For all statistical analyses, confirm that the following items are present in the figure legend, table legend, main text, or Methods section.

n/a	Confirmed	
☐	☒	The exact sample size (*n*) for each experimental group/condition, given as a discrete number and unit of measurement
☒	☐	A statement on whether measurements were taken from distinct samples or whether the same sample was measured repeatedly
☒	☐	The statistical test(s) used AND whether they are one- or two-sided<br>*Only common tests should be described solely by name; describe more complex techniques in the Methods section.*
☒	☐	A description of all covariates tested
☒	☐	A description of any assumptions or corrections, such as tests of normality and adjustment for multiple comparisons
☐	☒	A full description of the statistical parameters including central tendency (e.g. means) or other basic estimates (e.g. regression coefficient) AND variation (e.g. standard deviation) or associated estimates of uncertainty (e.g. confidence intervals)
☒	☐	For null hypothesis testing, the test statistic (e.g. *F*, *t*, *r*) with confidence intervals, effect sizes, degrees of freedom and *P* value noted<br>*Give P values as exact values whenever suitable.*
☒	☐	For Bayesian analysis, information on the choice of priors and Markov chain Monte Carlo settings
☒	☐	For hierarchical and complex designs, identification of the appropriate level for tests and full reporting of outcomes
☒	☐	Estimates of effect sizes (e.g. Cohen's *d*, Pearson's *r*), indicating how they were calculated

Our web collection on statistics for biologists contains articles on many of the points above.

Software and code

Policy information about availability of computer code

Data collection	CryoEM data was collected using EPU 2 software (Thermo Fischer Scientific).<br>X-ray crystallography data was collected with UDC.
Data analysis	CryoEM data processing was performed in MotionCor2, Relion 3.0 and Phenix 1.17. X-ray crystallography data was processed with Xia2 (apo) or AutoProc (ADP), and structure refinement was done in Phenix 1.17 and Isolde 1.2.2. Model building was done in Coot. Figures were illustrated using ChimeraX (UCSF), and PyMol (Schrödinger). All software is available free of charge for academic use.

For manuscripts utilizing custom algorithms or software that are central to the research but not yet described in published literature, software must be made available to editors and reviewers. We strongly encourage code deposition in a community repository (e.g. GitHub). See the Nature Research guidelines for submitting code & software for further information.

Data

Policy information about availability of data

All manuscripts must include a data availability statement. This statement should provide the following information, where applicable:

- Accession codes, unique identifiers, or web links for publicly available datasets
- A list of figures that have associated raw data
- A description of any restrictions on data availability

The map for the ParA-ATPgS-DNA cryo-EM structure was deposited in the EMDB, accession number EMD-12515. The corresponding atomic coordinates were deposited on the PDB, accession number 7NPF. The structure factors and atomic coordinates for the ParA2 apo and ADP-bound crystal structures were deposited in the PDB, with accession numbers 7NPD and 7NPE, respectively.
The molecular replacement for the ParA2 structure was performed using the P1 ParA crystal structure as a search model, accession number 3ZE6.

April 2020

Field-specific reporting

Please select the one below that is the best fit for your research. If you are not sure, read the appropriate sections before making your selection.

☒ Life sciences ☐ Behavioural & social sciences ☐ Ecological, evolutionary & environmental sciences

For a reference copy of the document with all sections, see nature.com/documents/nr-reporting-summary-flat.pdf

Life sciences study design

All studies must disclose on these points even when the disclosure is negative.

Sample size	The cryo-EM structure was derived from 182997 filament segments. The crystal structures were derived from 28,240 (apo), or 99,408 (ADP-bound) reflections, respectively.
Data exclusions	2D classification and 3D classification was applied during CryoEM data processing to select particles for high-resolution 3D refinement, as described in the online methods section. This is common practice to deal with heterogeneity.
Replication	Does not apply, as structure determination does not require replicates. This is common practice in the field.
Randomization	Does not apply, as data processing in cryo-EM or X-ray crystallography have internal measures (FSC crurves, Rfree) to avoid biaises.
Blinding	We did not blind data collection or analysis as these are not required for cryo-EM or X-ray crystallization analysis, as standard practice in the field.

Reporting for specific materials, systems and methods

We require information from authors about some types of materials, experimental systems and methods used in many studies. Here, indicate whether each material, system or method listed is relevant to your study. If you are not sure if a list item applies to your research, read the appropriate section before selecting a response.

Materials & experimental systems

n/a	Involved in the study
☒	☐ Antibodies
☒	☐ Eukaryotic cell lines
☒	☐ Palaeontology and archaeology
☒	☐ Animals and other organisms
☒	☐ Human research participants
☒	☐ Clinical data
☒	☐ Dual use research of concern

Methods

n/a	Involved in the study
☒	☐ ChIP-seq
☒	☐ Flow cytometry
☒	☐ MRI-based neuroimaging
